# Cultivation of *Chroococcidiopsis thermalis* Using Available In Situ Resources to Sustain Life on Mars

**DOI:** 10.3390/life14020251

**Published:** 2024-02-13

**Authors:** Giacomo Fais, Mattia Casula, Agnieszka Sidorowicz, Alessia Manca, Valentina Margarita, Pier Luigi Fiori, Antonella Pantaleo, Pierluigi Caboni, Giacomo Cao, Alessandro Concas

**Affiliations:** 1 Interdepartmental Centre of Environmental Science and Engineering (CINSA), University of Cagliari, Via San Giorgio 12, 09124 Cagliari, Italy; giacomo.fais@unica.it (G.F.); mattia.casula@unica.it (M.C.); a.sidorowicz@studenti.unica.it (A.S.); giacomo.cao@unica.it (G.C.); 2 Department of Mechanical, Chemical and Materials Engineering, University of Cagliari, Via Marengo 2, 09123 Cagliari, Italy; 3 Department of Biomedical Science, University of Sassari, Viale San Pietro, 07100 Sassari, Italy; alessia_manca@hotmail.it (A.M.); vmargarita@uniss.it (V.M.); fioripl@uniss.it (P.L.F.); apantaleo@uniss.it (A.P.); 4 Department of Life and Environmental Sciences, University of Cagliari, 09042 Cagliari, Italy; pierluigi.caboni@unica.it; 5 Center for Advanced Studies, Research and Development in Sardinia (CRS4), Loc. Piscina Manna, Building 1, 09050 Pula, Italy

**Keywords:** cyanobacteria, *Chroococcidiopsis thermalis*, in situ resource utilization, deep space exploration, astrobiology, crewed missions on Mars

## Abstract

The cultivation of cyanobacteria by exploiting available in situ resources represents a possible way to supply food and oxygen to astronauts during long-term crewed missions on Mars. Here, we evaluated the possibility of cultivating the extremophile cyanobacterium *Chroococcidiopsis thermalis* CCALA 050 under operating conditions that should occur within a dome hosting a recently patented process to produce nutrients and oxygen on Mars. The medium adopted to cultivate this cyanobacterium, named Martian medium, was obtained using a mixture of regolith leachate and astronauts’ urine simulants that would be available in situ resources whose exploitation could reduce the mission payload. The results demonstrated that *C. thermalis* can grow in such a medium. For producing high biomass, the best medium consisted of specific percentages (40%vol) of Martian medium and a standard medium (60%vol). Biomass produced in such a medium exhibits excellent antioxidant properties and contains significant amounts of pigments. Lipidomic analysis demonstrated that biomass contains strategic lipid classes able to help the astronauts facing the oxidative stress and inflammatory phenomena taking place on Mars. These characteristics suggest that this strain could serve as a valuable nutritional resource for astronauts.

## 1. Introduction

**Figure 1:**
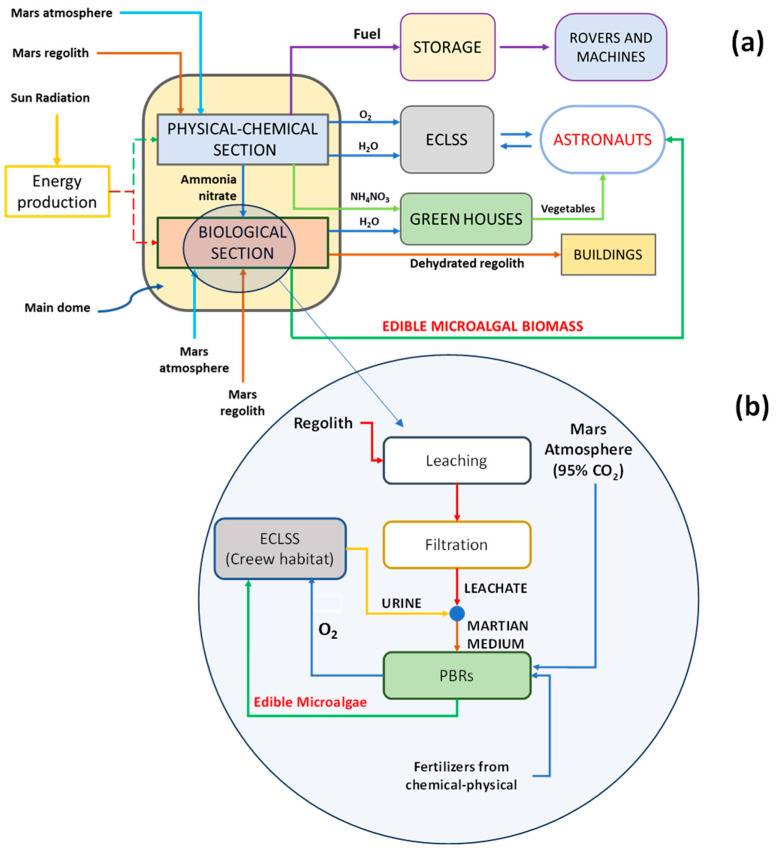
Scheme of the ISRU process to produce useful materials on Mars (**a**) and focus on the production of microalgae or cyanobacteria in the biological section (**b**). Adapted from Brughitta et al. [9].

The potential of colonizing distant celestial bodies, notably Mars, emerges as a prospective solution to humanity’s issues, such as overpopulation, climate change crisis, wars, pandemics etc. In this scenario, achieving full autonomy of future Martian colonies becomes imperative, given the intricacies and costs of resupply logistics that would arise from the huge distances separating Earth from extraterrestrial outposts [1]. This obstacle has led to a growing interest in the development of life support system technologies, including bioregenerative systems that can provide sustenance by relying also on in situ resource utilization (ISRU) [2]. Currently, most ISRU technologies rely solely on physical and chemical methods for producing oxygen and are unable to produce edible biomass. As a result, environmentally controlled life support systems (ECLSSs), which also utilize metabolic waste for food production by taking advantage of bioengineering technologies incorporating microorganisms, have been proposed. Among these, cyanobacteria and microalgae, i.e., oxygenic photosynthetic organisms, are particularly promising [3,4,5]. In this regard, Cao et al. [6] recently filed a patent regarding an ISRU process, which also involves microalgae and cyanobacteria, that would ensure food and supplements production for astronauts using resources available on Mars, such as Martian soil, known as regolith, human metabolic waste products, such as astronaut urine, and the naturally occurring CO_2_ in the Martian atmosphere or within a crew-inhabited habitat [7,8]. In the proposed process, aimed at generating suitable quantities of biomass to meet the requirements of crew, a chemical–physical section is integrated with a biological one to produce water, oxygen, food and supplements through the synergistic utilization of resources available on Mars, metabolic waste, and a necessary minimal amount of materials supplied from Earth (Figure 1a) [6,7].

Noteworthily, food and supplement production occurs within photobioreactors through the cultivation of microalgae or cyanobacteria (Figure 1b), which are supplied with a mixture of regolith leachate, astronauts’ urine, and trace metals brought from Earth. The cultivated microalgae should meet the crew’s dietary requirements and contribute to oxygen regeneration for cabin air supply. Moreover, the adoption of this approach would result in reduced payload demands, thereby enhancing the feasibility of long-term missions. Microalgae could be cultivated using various photobioreactor configurations, such as open pond systems located indoors within pressurized domes, as illustrated in the Graphical abstract and detailed in the process by Cao et al. [6]. This design would require a manageable payload, including a liner and a pump. It is important to note that this approach assumes that the domes can provide adequate protection against radiation and maintain the necessary temperature as well as the desired light duration for photosynthetic growth. Various designs and materials have been proposed in the existing literature for inflatable domes on Mars that enable the growth of cyanobacteria and microalgae [10,11,12,13,14].

The crucial issue is that the organic biomass produced by these microorganisms is typically rich in bioactive compounds, such as polyphenols or antioxidant pigments, and possesses high nutritional value. This makes it a promising candidate for utilization as a food source or dietary supplement for astronauts involved in missions to Mars. Among these strains, *Spirulina* spp. have received the most comprehensive examination for this purpose [15,16,17]. Furthermore, some photosynthetic microorganisms, designated as poly-extremophiles due to their capability to endure a wide range of harsh environmental conditions, have been suggested for use in space technologies. For instance, in recent years a cyanobacterium known as *Chroococcidiopsis* has gained increasing attention in the framework of space missions [18,19,20]. This species is among the most primitive genera of photosynthetic and extremophile cyanobacteria on Earth [21]. *Chroococcidiopsis* spp. is considered the most desiccation-resistant cyanobacterium and is found in extremely arid habitats. The strain C. *thermalis* displays high Rubisco enzyme specificity for CO_2_ and catalytic carboxylation efficiency, along with the most effective CO_2_-concentrating mechanisms among cyanobacteria, thus enabling its survival in CO_2_-limited environments such as elevated temperatures and/or desert areas [22]. Moreover, its distribution extends to a variety of other harsh ecological niche, including Antarctic rocks, thermal springs, and hypersaline habitats. Several scientific studies describe this cyanobacterium able to survive in extreme conditions such as extreme temperatures, long-term desiccation, nutrient deprivation, extreme pH values, high perchlorate concentration or high radiation [23,24,25,26]. Due to these interesting feature, *Chroococcidiopsis* spp. are a candidate to survive beyond the Earth. Additionally, the endolithic character of its growth has been suggested as a model for the development of technologies for large-scale cultivation on Mars regolith [27]. As for its use to produce food for astronauts, its biomass can be a source of proteins, lipids, polysaccharides, pigments (such as chlorophylls, carotenoids, phycobiliproteins) phenolic compounds, and vitamins [28,29,30].

In a recent study under conditions resembling those of Mars, the capability of *Chrococcidiopsis* sp. CCMEE 029 to tolerate perchlorate salts, which are commonly found in Martian regolith, was examined, suggesting its adaptability to perchlorates and indicating that this strain can survive in the presence of such toxic compounds [24]. On the other hand, it is fundamental to take into account the potential variation in the composition of Martian regolith depending on the specific geographical location of the regolith [31]. The simulant we used in this work is representative of a Mars zone where perchlorates are not in the regolith. Moreover, there are various technologies that have the potential to eliminate perchlorates from regolith, which could make Martian soils, initially containing these hazardous compounds, suitable not only for the cultivation of microalgae and cyanobacteria but also for growing plants [32]. For instance, Devila et al. [33] have proposed a method for perchlorate removal from Martian soil, which could also serve for oxygen generation, both for human consumption and for supporting surface operations [33]. Therefore, Martian regolith represents a valuable resource with multiple potential applications during space missions and planetary exploration. It can be utilized for constructing habitats, producing oxygen, and extracting water, thus providing essential resources for astronauts [34]. Additionally, a relevant aspect in the context of this research is that Martian regolith can be employed for cultivating plants and microorganisms, as it contains minerals and valuable resources for scientific and industrial purposes [35,36,37,38,39].

In this regard, the use of Martian regolith as a mineral source for the growth of cyanobacteria and microalgae has already been proposed, primarily investigating the feasibility of utilizing regolith to obtain growth medium. However, the aim of these studies was not to achieve high biomass production yields to meet the needs of a manned mission to Mars, but rather to investigate the microorganisms’ ability to survive in growth media produced by exploiting regolith. For instance, Olsson-Francis et al. employed a lithotrophic medium in which extremophilic cyanobacteria, including *Chroococcidiopsis* spp., exhibited mere survival [35]. In a study conducted by Ramalho et al. [40], it was suggested that leachates obtained from only regolith simulants do not ensure a substantial production of microalgal biomass. Within the same study, the cyanobacterium Anabaena sp. PCC 7938 demonstrated lower productivity compared to the control cultivated in BG11 medium (approximately 50% less). Similarly, Macario et al. [41] reported an increase in optical density when species were cultivated in synthetic media. *N. muscorum* and *A. cylindrica* demonstrated their ability to survive when cultivated in an extract of Martian regolith, albeit with a lower biomass yield compared to growth in the standard medium. *A. platensis* exhibited minimal growth. In the same context, Fernandez et al. [42], demonstrated that cultures of *Chroococcidiopsis* sp. exhibited enhanced growth when exposed to a lunar regolith simulant compared to a Martian regolith simulant, producing approximately 50% and 30% of the biomass produced in the classic BG11 medium, respectively.

To address this need and to investigate the feasibility of utilizing in situ resources on Mars for cultivating photosynthetic organisms and producing food to supplement astronauts’ nutritional requirements, our recent work has revealed that a cyanobacteria *Spirulina* sp. and *Synechococcus* sp. can utilize simulated Martian atmosphere to produce edible biomass through photosynthesis. Furthermore, the investigated cyanobacteria were cultivated using a growth medium composed of a mixture of a simulant of Martian regolith leachate and a human urine simulant [7,8]. In this scenario, this study aims to evaluate the feasibility of cultivating the polyextremophile cyanobacterium *Chroococcidiopsis thermalis* using in situ resources available on Mars such as Martian regolith and human urine. The main objective is to assess the impact of these resources on the growth and on the composition of *C. thermalis*, with particular attention given to its nutritional profile and antioxidant power. Additionally, the study investigated for the first time the cytotoxicity of the produced biomass through an appropriate in vitro test.

The findings of this research could lead to new developments in the cultivation of crucial resources in extraterrestrial environments to sustain astronauts during long-term missions to Mars and to cultivate cyanobacteria species under extreme conditions.

## 2. Materials and Methods

### 2.1. Chemicals

Eur.-Reag grade chemicals were procured from Sigma-Aldrich (Merck KGaA, Darmstadt, Germany), including sulfuric acid (96%), orthophosphoric acid (85%), sodium nitrate, potassium chloride, phenol, copper sulphate, sodium hydroxide, and sodium potassium tartrate. Similarly, grade RPE-ACS-for analysis-reag. Ph. eur.-reag chemicals were obtained from Carlo Erba (Valde Reuil Cedex, France), while sodium carbonate and Folin-Ciocalteau reagent were purchased from Sigma-Aldrich Inc. (St. Louis, MO, USA). Glucose, bovine serum albumin, and vanillin standards were acquired from Sigma-Aldrich (Merck KGaA, Darmstadt, Germany). Ultrapure water, with a conductivity below 18.2 MΩ, was obtained via distillation using a Milli-Q system (Millipore, Milan, Italy). Methoxyamine hydrochloride, NO-bis (trimethylsilyl) trifluoroacetamide were used for the derivatization. SPLASH^®^ LIPIDOMIX^®^ standard component mixture was purchased from Sigma Aldrich (Milan, Italy): PC (15:0–18:1) (d7), PE(15:0–18:1) (d7), PS (15:0–18:1) (d7), PG (15:0–18:1) (d7), PI (15:0–18:1) (d7), PA (15:0–18:1) (d7), LPC (18:1) (d7) LPC 25, LPE (18:1) (d7), Chol Ester (18:1) (d7), MG (18:1) (d7), DAG (15:0–18:1) (d7), TAG ((15:0–18:1) (d7)-15:0)), SM (18:1) (d9), Cholesterol (d7).

### 2.2. Preparation and Composition of the Martian Medium (MM)

In what follows, a brief summary of the experimental procedure adopted to obtain the Martian medium (MM) is described. MM was prepared by mixing a leachate of Martian regolith simulant (JSC MARS-1) and synthetic human urine (MP-AU) to simulate astronauts’ metabolism [43,44]. All components of this medium were prepared in the same method described in previous works [7]. MM and its dilutions with Z-medium have been sterilized at 121 °C for 21 min prior to use. As shown in Table 1, the composition of pure MM is adapted from the work by Concas et al. [8].

### 2.3. Culture Conditions and Growth Experiments to Optimize MM Content for Cultivation Purposes

*Chroococcidiopsis thermalis* Geitler (CCALA 050) was obtained from the Culture Collection of Autotrophic Organisms (CCALA). The strain was cultivated at 20 ± 1 °C under 12:12 light–dark illumination of 50 μmol photons m^−2^ s^−1^ (light meter delta) white light and continuous agitation at 50 rpm (Stuart SSM1, Biosigma orbital shaker) in Z-medium [45].

The growth experiments were executed in triplicate using a mixture composed of varying volume percentages of MM and Z-medium, i.e., 0%, 20%, 40%, 60% and 80% MM, respectively. The batch culture experiments were performed in 75 cm^3^ rectangular transparent vented cap flasks (Corning^TM^) filled to a volume of 40 mL and maintained under an illumination of 50 μmol photons m^−2^ s^−1^ on the irradiated surface of the culture flask. The culture pH was measured daily using a pH-meter (Basic 20, Crison), while the growth was monitored by quantifying the chlorophyll-a optical density (OD) of the culture at 650 nm using a Genesys 20 spectrophotometer (Thermo Scientific, Waltham, MA, USA).

**Figure 2:**
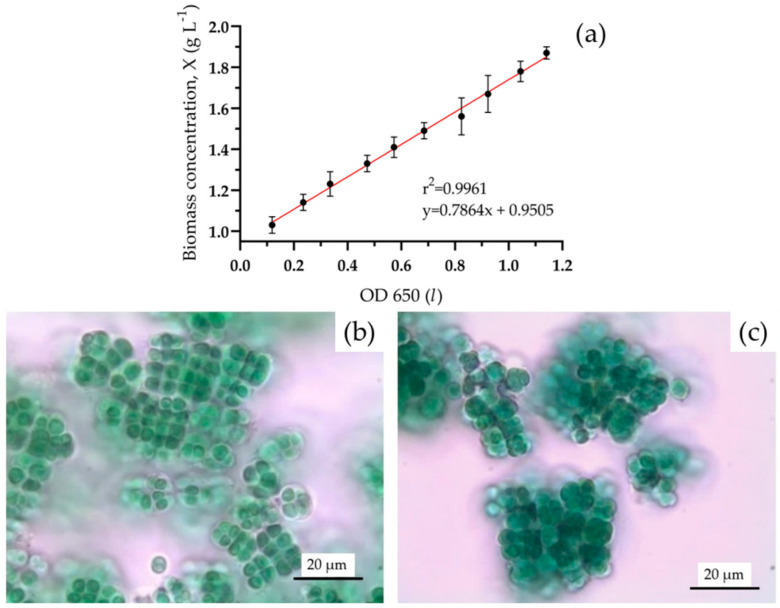
Calibration line C_x_ vs. OD for *C. thermalis* (**a**); *C. thermalis* cultivated in MM0 (**b**) and in MM40 (**c**), scale bar = 20 µm.

The biomass concentration (C_x_, g L^−1^) was calculated from the OD measurements using a calibration curve (C_x_ vs. OD) as depicted in Figure 2a. The calibration line was established by gravimetric analysis of the biomass concentration of known culture volumes that were previously centrifuged at 4000 rpm for 15 min and lyophilized with LIO-5PDGT freeze-dryer (5 pascal, Milano, Italy).

Cell morphology (Figure 2b,c) was investigated using an optical microscope (Leica DM750) interfaced with a Leica EC3 digital camera (Leica Microsystems, Wetzlar, Germany), and the Leica Application Suite (version 3.4.0, Leica Microsystems).

### 2.4. Sample Preparation for Chemical Characterization Analysis

An aliquot of *C. thermalis* culture was centrifuged at 4000 rpm for 10 min at 20° C. The supernatant was eliminated, and the pellet was resuspended in Milli-Q water. The washing procedure was repeated three times. The cellular pellet was then frozen at −80 °C, lyophilized with an LIO-5PDGT freeze-dryer and finally pulverized with mortar and pestle. The dried powder was stored in darkness inside a glass vacuum desiccator before the analytical procedure.

### 2.5. Determination of Total Carbohydrates, Lipids, and Total Soluble Proteins

The quantification of macronutrients was performed according to the methodology utilized in the study of Concas et al. [8]. The analysis of carbohydrates (C) was executed using the method described by Dubois et al. [46], while the determination of total lipids (TL) was performed by means of the techniques established by Chen and Vaidyanathan [47] and Bligh and Dyer [48]. The protocol by Lowry et al. [49] was utilized to determine the concentration of total proteins (TSP). The samples were analyzed using a Varian Cary 50 spectrophotometer and quantified using the external standard method. The results are reported as means ± standard deviation and expressed in mg/kg, with all samples being analyzed in triplicate.

### 2.6. Determination of Fatty Acid Methyl Esters (FAMEs) Using GC-FID Analysis

Total lipids were extracted using a modified Folch method [50]. Briefly, 10 mg of lyophilized biomass was extracted using a mixture of chloroform/methanol (2:1 *v*/*v*) and sonicated three times. Then, a volume of KCl 0.2M was added to improve the extraction. The solution was centrifuged at 4000 rpm for 20 min and then the lipid phase was dried under a gentle nitrogen stream. Fatty acids methyl esters were obtained by performing a transesterification with sodium methoxide at 25% (*w*/*v*). Briefly, the dried lipid phase was reconstituted by using hexane and then by adding sodium methoxide. The samples were heated at 55 °C for 30 min. Then a solute of HCl 1N was added. The supernatant was filtered using a 0.45 μm filter and injected into the GC-FID system. After that, FAME extracts were injected into the GC system (8860 GC system, Agilent, Santa Clara, CA, USA) equipped with a flame ionization detector (FID) and a fused capillary column Agilent HP-5 (30 m × 0.32 i.d, 0.25 μm f.t.). The injector and detector temperatures were set at 260 °C, and the carrier gas (nitrogen) flow was 1 mL/min. The temperature of the oven was held at 50 °C for 1 min before being increased from 50 to 175 °C at 10 °C/min, held at 175 °C for 10 min, from 175 to 210 °C at 5.0 °C/min, held at 210 °C for 10 min, increased from 210 °C to 230 °C at 5.0 °C/min, held at 230 °C for 9.5 min, and finally increased from 230 °C to 300 °C at 10 °C/min. The sample was injected in split mode (0.4 μL) with a split ratio set at 1:20. Peak identification was done by comparing peak retention time with Supelco 37 component FAME Mix (Sigma Aldrich). Data are expressed as a g/100g of dried biomass (mean ± standard deviation).

### 2.7. Lipidomic Analysis

A modified Folch method was used to extract the total lipids of twelve lyophilized samples [50]. Briefly, 1 mg of each lyophilized biomass sample was transferred to an Eppendorf tube containing 10 µL of the internal mixture of standards (Splash, Lipidomics, Sigma Aldrich, Milan, Italy) and then a mixture of chloroform: methanol (2:1 *v*/*v*) was added. The solution was sonicated for 30 min at 4 °C. Finally, 90 μL of aqueous 0.2 M potassium chloride were added. The suspension was then centrifuged at 14.000× *g* for 10 min at 4 °C. After centrifugation, the lipophilic layer was transferred to a glass vial and dried by a gentle nitrogen stream. The dried phase of the cyanobacteria extracts was reconstituted using 20 µL of a mixture of methanol–chloroform (1:1 *v*/*v*) and diluted with 980 µL of a mixture of 2-propanol–acetonitrile–water (2:1:1 *v*/*v*/*v*). Then, the samples were analyzed using a UHPLC-QTOF/MS coupled with an Agilent 1290 Infinity II LC system, injecting 1 μL and 5 μL in the positive and negative ionization mode, respectively. Chromatographic separation of lipids was obtained with a Kinetex 5 µm EVO C18 100 A, 150 mm × 2.1 μm column (Agilent Technologies, Palo Alto, CA, USA). The column was maintained at 50 °C at a flow rate of 0.4 mL/min. The mobile phase for positive ionization mode consisted of a mixture of solvent A (10 mM ammonium formate solution in 60% of milli-Q water and 40% of acetonitrile) and solvent B (10 mM ammonium formate solution containing 90% of isopropanol, 10% of acetonitrile). In positive ionization mode, the chromatographic separation was obtained with the following gradient: initially 60% of A, then a linear decrease from 60% to 50% of A in 2 min then at 1% in 5 min while keeping this percentage for 1.9 min and then brought back to the initial conditions in 1 min. The mobile phase for negative ionization mode differed only for the use of 10 mM ammonium acetate instead of ammonium formate. The Agilent jet stream source was operated with the following parameters: gas temperature, 200 °C; gas flow (nitrogen) 10 L/min; nebulizer gas (nitrogen), 50 psig; sheath gas temperature, 300 °C; sheath gas flow, 12 L/min; capillary voltage 3500 V for positive and 3000 V for negative; nozzle voltage 0 V; fragmentor 150 V; skimmer 65 V, octapole RF 7550 V; mass range, 50−1700 *m*/*z*; capillary voltage, 3.5 kV; collision energy 20 eV in positive and 25 eV in negative mode, mass precursor per cycle = 3; threshold for MS/MS 5000 counts. Before the analysis, the instrument was calibrated using an Agilent tuning solution at the mass range of m/z 50–1700. Samples were acquired in an auto MS/MS method using an iterative mode to reveal the maximum number of lipid species with a mass error tolerance of 20 ppm with a retention exclusion tolerance of 0.2 min. The Agilent MassHunter LC/MS Acquisition console (revision B.09.00) and lipid annotator from the MassHunter suite were used for data acquisition and data processing. Lipid levels were normalized using the following internal standard PE 33:1 (d7) for positive and negative ionization modes, respectively. Mass spectrometer mass accuracy expressed as error in parts per million was calculated with Equation (1) [51]:

(1) $$ppm=\frac{{\left(m/z\right)}_{theo}-{\left(m/z\right)}_{exp}}{{\left(m/z\right)}_{theo}}{\cdot 10}^{6}$$

where ${\left(m/z\right)}_{theo}$ is the theoretical value of mass-to-charge ratio while ${\left(m/z\right)}_{exp}$ is the corresponding experimental value.

### 2.8. Chlorophyll-a, Total Carotenoids, Phycocyanin and Allophycocyanin

The content of chlorophyll-a (Chl) and total carotenoids (TCs) was determined using the method by Zavrel et al. [52]. 1000 µL of culture were centrifuged at 10,000 rpm for 10 min at 4°C, then the supernatant was discarded, and 1 mL of neutralized methanol was added to the pellets. The samples were left overnight in the fridge and then homogenized using three cycles of 10 min of vertexing and ultrasonic bath. The solutions were then centrifuged for 10 min at 10,000 rpm and the supernatant analyzed spectrophotometrically for chlorophyll-a and total carotenoids at λ = 720 nm and λ = 665 nm, respectively, with methanol as a blank at λ = 470 nm. The concentrations were estimated using the correlations proposed by Ricthie [53] for total carotenoids and Wellburn [54] for chlorophyll-a. Then, the procedure outlined by Lobban et al. [55] was utilized to perform the analysis of phycobiliproteins (phycocyanin (P) and allophycocyanin (APC)). 1000uL of a 0.1 M PBS with a pH of 7 were added to the pellets and subjected to ultrasonic treatment for two hours at 30 °C in a water bath. The resulting mixture was then centrifuged at 14,000× *g* for 10 min, and the supernatant was transferred to a clean Eppendorf tube. The absorbance for each sample was measured at 565 nm, 615 nm, 652 nm, and 720 nm and the concentration of phycobiliproteins was determined by Bennet and Bogorad equations [56].

### 2.9. Antioxidant Power Determination

The method described by Brand-Williams et al. was employed in this study [57]. To prepare the sample, 2 mg of lyophilized *Chroococcidiopsis* powder was mixed with 250 μL of methanol and 100 mg of 1–1.3 mm glass balls, and the resulting solution was vortexed and sonicated in an ultrasonic bath for 10 min at 10 °C and repeated three times. After centrifugation at 4000 rpm for 5 min, 50 μL of the supernatant obtained from the methanol extract was mixed with 2 mL of a methanol solution containing 50 μmol of DDPH to determine the total or standard polyphenol levels. The mixture was then incubated for 60 min and the solutions were analyzed at λ = 517 nm. The quantitative analysis was performed using the external standard method (Trolox), which involved correlating the absorbance with the concentration. The results are reported in mmol/kg TEAC (Trolox equivalent antioxidant capacity).

### 2.10. Preparation of Chroococcidiopsis Culture Extracts for MTT Assay

The culture of *C. thermalis* was analyzed when the optical density at 650 nm reached values close to 1. To harvest fresh biomass and collect supernatant, a modified protocol previously reported by Hernandez et al. was used [58]. Cyanobacteria in the exponential phase of growth, both in Z medium (MM0) and MM40, were centrifuged at 5.000× *g* for 20 min, and the supernatant was collected and sterilized by filtration through a 0.22 µm filter (Merck, Millipore). The pellet was resuspended in 70% ethanol (10 mL of solvent for 1 g of fresh biomass), and after 2 h, the solution was centrifuged at 12.000× *g* for 15 min. The supernatants were collected, and the solvent was evaporated at 37 °C. The dry residues were resuspended in RPMI 1640 medium (Sigma Chemical Co., Milan, Italy) supplemented with 10% inactivated fetal bovine serum (FBS), 100 IU/mL penicillin, and 100 μg/mL streptomycin and sterilized by filtration with a 0.22 µm filter.

### 2.11. MTT Assay for In Vitro Cytotoxicity Activity

Human epithelial carcinoma cell lines, HeLa, cultured in RPMI 1640 medium supplemented with 10% FBS, 100 IU/mL penicillin, and 100 μg/mL streptomycin, at 37 °C in a 5% CO_2_ atmosphere, were used to assess the cytotoxicity of supernatant and cyanobacterial extracts. The cells were seeded in 96-well microplates at 1.5 × 10^4^ cells/well with overnight incubation at 37 °C. When a confluent monolayer was formed, 100 µL of fresh medium containing serial twofold dilutions of supernatants and serial dilutions of the extract ranging from 60.00 mg/mL to 3.75 mg/mL were added, and cells were incubated for 24 h. Cell viability was investigated by the MTT (3-(4,5-dimethylthiazol-2-yl)-2,5-diphenyltetrazolium bromide) assay as previously described [59]. Proliferation activity of sample-treated cells was detected by measuring the absorbance at 570 nm using a spectrophotometer and was expressed as a percentage of cell viability compared to control (100%). The results of experiments (performed in triplicate) are expressed as the mean values ± standard deviation.

### 2.12. Statistical Analysis

At least three independent repetitions of each experiment were performed, and the corresponding results are reported in terms of means ± SD. Statistical analyses were performed using GraphPad PRISM 7.00 (GraphPad Software, San Diego, CA, USA). The means and standard deviation (SD) were calculated from three independent experiments, each consisting of triplicate analyses for each sample/condition. Data analysis involved ANOVA and Dunnett’s and Sidak’s tests to compare multiple groups with the control group, while controlling for cumulative alpha error. The significance levels based on the *p*-values are indicated by asterisks (*). No asterisk denotes *p*-value > 0.05; * *p*-value < 0.05; ** *p*-value < 0.005; *** *p*-value < 0.0005; and **** *p*-value < 0.0001.

## 3. Results and Discussion

### 3.1. Optimization of the Martian Culture Medium MM

**Figure 3:**
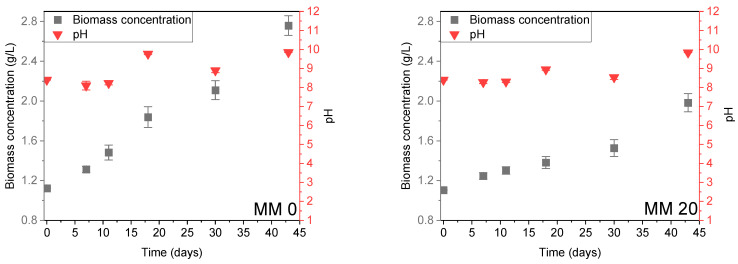

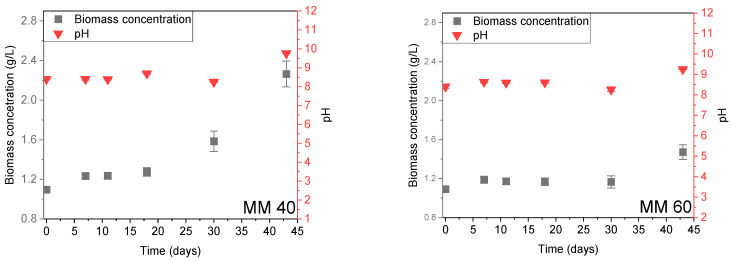
The effect of Z-medium volume replacement with Martian medium (MM) at 0%, 20%, 40%, and 60%.

Growth experiments of *Chroococcidiopsis thermalis* in different dilutions of Martian medium (MM) were carried out for 43 days and the corresponding time evolution of biomass concentration and pH is shown in Figure 3.

The strain was cultivated in a pure Z-medium (MM0), resulting in an increase in initial concentration from 1.12 ± 0.01 g L^−1^ to 2.76 ± 0.01 g L^−1^ (Figure 3, MM0). The experiment was then modified to evaluate the suitability of in situ Mars resource utilization by replacing 20% of the MM0 volume with a corresponding volume of MM. Such a replacement led to the onset of a relevant lag phase indicating an inhibitory effect on *C. thermalis* and lasting about 20 days during which the growth rate was very low, and the pH remained almost constant (Figure 2, MM20). However, after 25 days the strain managed to adapt to the new medium starting the exponential phase and achieving biomass concentrations only slightly lower than the ones observed with Z-medium (MM0). Further trials were conducted by replacing 40% (Figure 2, MM40) and 60% (Figure 2, MM60) of the MM0, respectively, with corresponding volumes of MM. In the case of MM40, a growth trend like the one already described for MM20 was observed, but at the end of the experiment (45 days), a biomass concentration close to the one observed using only Z-medium was achieved. This indicates that, albeit taking a very long time, the adaptation of the strain to the new growth medium was quite effective. This is a typical behavior of extremophile strains according to the literature [60,61]. The replacement of 60% of Z-medium with MM (Figure 3, MM60) led to a substantial inhibition of the culture to the point where, once the exponential phase took place, only a slight increase of the concentration was observed with respect to the initial one.

**Figure 4:**
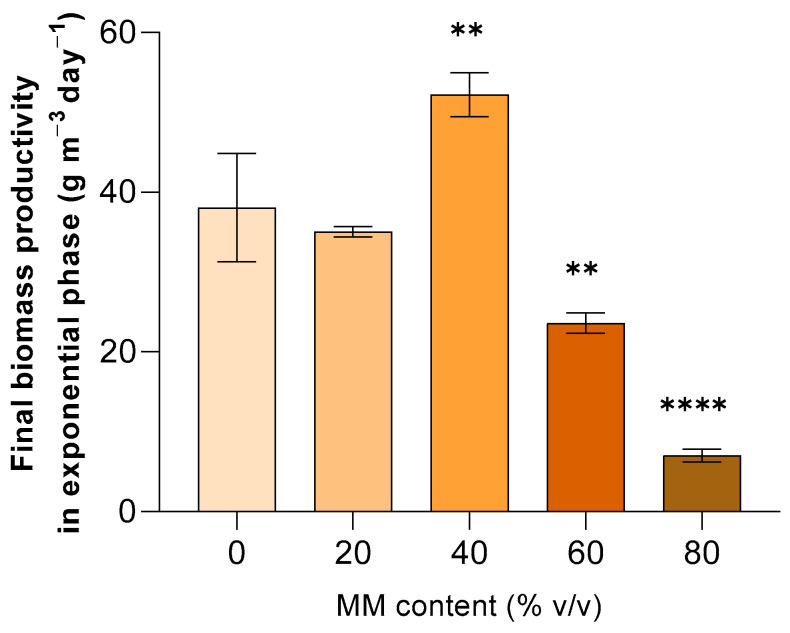
Effect of MM content in the batch growth medium on the final biomass productivity in exponential phase after 43 days of cultivation. Mean differences were tested using one-way ANOVA. No asterisk denotes *p*-value > 0.05; ** *p*-value < 0.01; **** *p*-value < 0.0001.

Figure 4 shows the biomass productivities achieved during the exponential growth phase. It can be observed that the use of MM40 led to a minor impact on culture evolution, with a long latency phase but a higher final biomass productivity in exponential phase of 52 ± 2.70 g m^−3^ day^−1^ that is even greater than the one (38.1 ± 6.90 g m^−3^ day^−1^) of the pure MM0 culture (Table S1 of Supplementary Materials).

However, by further increasing the MM content to 60%vol (MM60), a substantial decline in overall growth rate and thus biomass productivity was observed, even in the exponential phase. In fact, replacing 60% of MM0 with pure MM resulted in a significant inhibition of the growth probably due to too high an osmotic stress. Indeed, according to the literature, perturbations in osmotic balance can adversely affect cyanobacteria even if at specific levels it can be tolerated and may even elicit physiological responses that enhance the production of specific compounds [62,63]. The salinity levels of MM40 are probably close to the optimal ones for the growth of *C. thermalis*, while that of MM60 culture (520 ± 3 µS cm^-1^) is too high as a result of the high salt concentration in the human urine simulant. It should be noted that, according to the literature, the high salinity of MM richer media can be the cause of the long lag phase observed in the same media [64].

Ultimately, based on the results above, the medium MM40 represented the most favorable trade-off between biomass productivity and in situ resources utilization that corresponds to reduction in the payload. In light of the above, we performed the following chemical and biochemical analyses only on the biomass from MM40 and ZM because other relevant changes occurred in the biochemical composition and nutritional value of *C. thermalis* as a result of the use of a medium that might be produced in situ.

### 3.2. Analysis of the Macronutrient’s Composition of Chroococcidiopsis Thermalis Biomass

**Figure 5:**
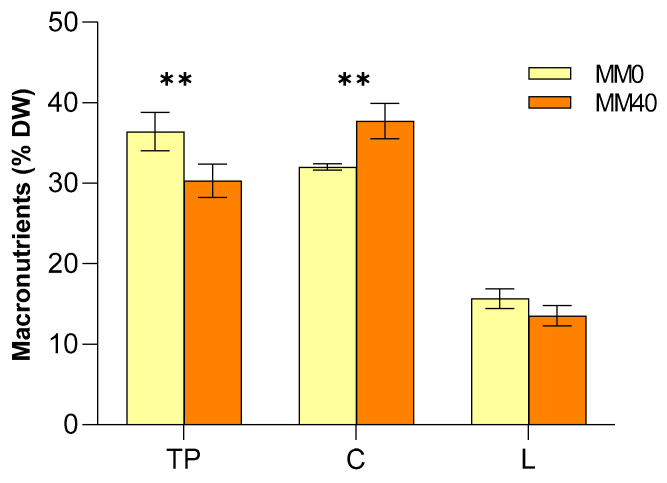
Effect of MM40 on the macronutrient (% DW) after 43 days of cultivation. Total protein (TP), carbohydrate (C), and total lipid (TL). Mean differences were tested using two-way ANOVA. No asterisk denotes *p*-value > 0.05; ** *p*-value < 0.01.

Astronauts have special nutritional needs due to the unique space conditions and physical demands. Providing them with a balanced and highly nutritious diet is essential for their health and performance during long periods of intense work in a reduced gravity environment [65]. In this perspective, astronauts require an adequate protein intake to support muscle mass and recovery, as well as carbohydrates and lipids to provide energy during space activities [66,67,68]. For this reason, to determine the suitability of the produced biomass as a dietary supplement, the macronutrient profile, including proteins (TP), carbohydrates (C), and lipids (TL), were first analyzed. The results reported in Figure 5 showed that the biomass was composed of TP, with a content of 36.39 ± 2.48% (DW), while C and TL were present in the ranges of 32.02 ± 0.44% and 15.66 ± 1.22%, respectively, for MM0. As for MM40, values of 30.28 ± 2.08%, 37.73 ± 2.23% and 13.47 ± 1.26% were correspondingly obtained for TP, C and TL, respectively. The obtained results suggest that the biomass of *C. thermalis* cultivated in a medium potentially producible in situ (MM40) could provide a sustainable source of macronutrients for astronauts’ diets. Indeed, the calculated potential caloric intake from MM40 biomass composition according to the procedure proposed by the FAO [69] is about 393.27 calories for 100 g of dry powder. These values would permit meeting good percentages of the caloric need of astronauts with a few small bars of *C. thermalis* biomass while simultaneously reducing the space food system mass [70].

Raw data used for Figure 5 are reported in Table S2 of Supplementary Materials.

### 3.3. Fatty Acid Methyl Esters (FAMEs)

Gas chromatography coupled with mass spectrometry was used to analyze the composition of fatty acid methyl esters (FAMEs) in the two different growth media. The results reported in Table A1 (Appendix A) show that MM40 shows a higher percentage of palmitic acid (24.0 ± 0.1%) compared to MM0 (10.4 ± 0.10%). In addition, MM40 displays a higher percentage of palmitoleic acid (3.07 ± 0.03%) compared to MM0 (2.02 ± 0.02%).

However, MM0 his characterized by a higher percentage of linoleic acid (n-6) (3.3 ± 0.05%) compared to MM40 (1.8 ± 0.05%). As for the total saturated acids, MM40 has a higher content at 32.02% compared to MM0 at 18.75%. As for the monoenes, MM40 also shows a higher content at 7.76% compared to MM0 at 4.46%. *C. thermalis* grown in MM0 reveals a higher total content of PUFAs (5.16%) than MM40 (4.53%). Finally, the biomass obtained from cultivation in MM40 has a higher S/U ratio (2.60) when compared to MM0 (1.95).

**Figure 6:**
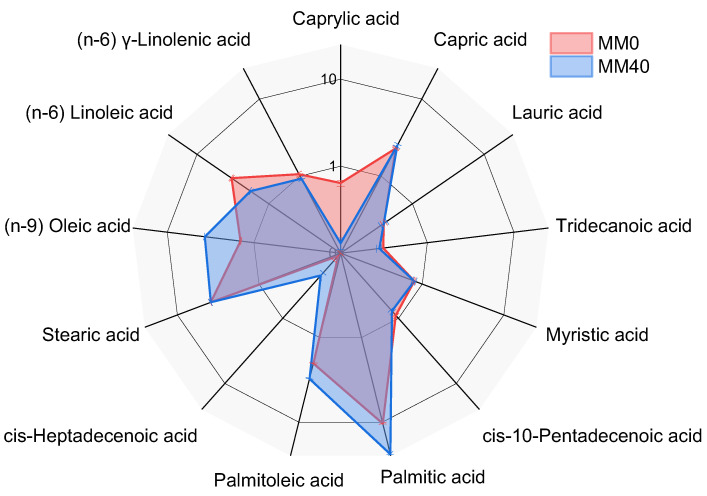
Comparison of MM40’s FAME composition with MM0, expressed as the logarithm (base 10) of the percentage composition of dried biomass.

The change in terms of FAMEs between algae grown in MM40 and MM0 are graphically highlighted in Figure 6 and may stem from different factors that could have significant implications on the nutritional value of biomass production in space.

Of particular concern is the higher concentration of palmitic acid, a saturated fatty acid, found in the biomass grown in MM40. It is widely known that palmitic acid has been linked to an increased risk of cardiovascular disease. However, under normal human physiological conditions, the body prevents the accumulation of palmitic acid by increasing delta 9 desaturation, which produces palmitoleic acid (16:1n-7, POA), elongating it to stearic acid (SA), and further converting it into oleic acid (18:1, OA) [71,72].

The average intake of palmitic acid is around 20–30 g/d, and the tight homeostatic control of its concentration in tissues is likely related to its fundamental physiological role in a number of biological functions [73]. Consequently, considering that the consumption of microalgae or cyanobacteria should not be a replacement for a normal and varied diet but rather a dietary integration, the daily average intake threshold of palmitic acid is not exceeded.

On the other hand, capric acid is a medium-chain FAME that has potential health benefits such as antimicrobial and antiviral properties. The higher concentration of capric acid in MM40 could be advantageous for space exploration as it could potentially help prevent infections. Furthermore, it also shows anti-inflammatory effects, can improve cognitive function, and has potential anticancer effects [74].

The higher content of total saturated FAMEs in MM40 compared to MM0 could be due to differences in the composition of the two growth media. In fact, the regolith leachate and, in particular, human urine, contain significant levels of metals (as indicated in Table 1) that could promote the synthesis of saturated fatty acids in microalgae. Particularly relevant in this regard is the presence of iron in the Martian regolith. In fact, the literature confirms that the increased iron (Fe) concentration in the growth medium results in the increase of saturated FAMEs in *Nannochloropsis occulata* and *Auxenochlorella protothecoides* and *C. vulgaris* SAG 211-12 [75,76,77].

Regarding total monoenes, the higher content in MM40 compared to MM0 could be the result of the different metals’ composition in the two growth media. Monounsaturated fatty acids are typically synthesized in response to specific nutrient starvation, and it is possible that the different chemical composition in MM40 may have influenced the fatty acid composition of the cyanobacterium. This class of FAMEs, which also includes oleic acid, is important for positively modulating various metabolic functions. MUFAs are also capable of altering plasma lipids and lipoprotein composition, thereby reducing inflammation, oxidative stress, and coagulation, and improving glucose homeostasis and blood pressure [74]. Overall, the results in Table A2 and Figure 6 suggest that the composition of the growth medium can significantly affect the composition of all classes of FAMEs in *C. thermalis*, with important implications for their potential use as a nutrient supplements in space. In this regard, further research is needed to optimize the growth conditions of cyanobacteria using Martian resources and to understand their impact on the better fatty acid composition.

### 3.4. Results from Lipidomic Analyses

The lipids of *C. thermalis* (Table A2, Appendix A) belong to several lipid classes, including triacylglycerol (TG), monogalactosyldiacylglycerols (MGDG), digalactosyldiacylglycerols (DGDG), sulfoquinovosyldiacylglycerols (SQDG), phosphatidylcholine(PC) and phosphatidylglycerols (PG). These findings are consistent with previous studies that have also examined the lipid composition of different microalgae and cyanobacterial cells [28,30,78,79,80]. The analysis showed notable variations in the levels of lipid species between *C. thermalis* cultivated in MM0 and MM40, as presented in Table A3 (Appendix A).

**Figure 7:**
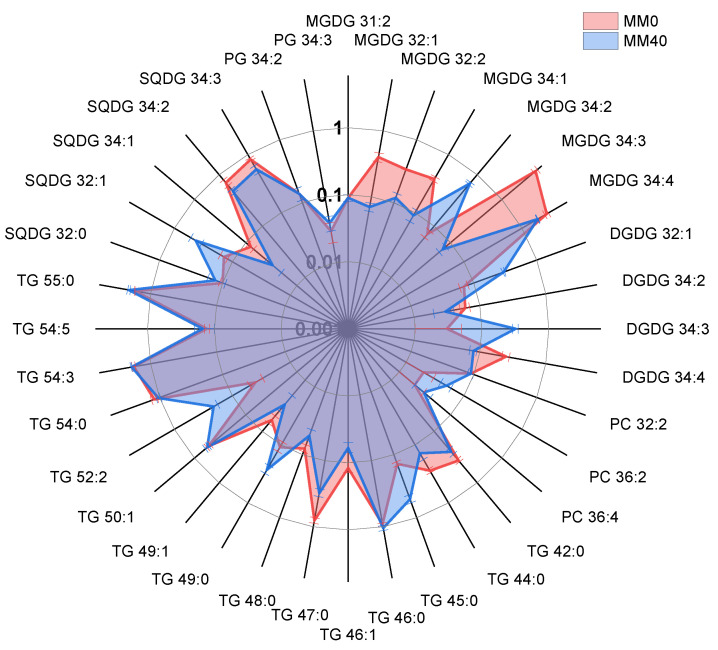
Comparison of MM40’s lipid composition with MM0, expressed as the logarithm (base 10) of the normalized lipid abundance of dried biomass.

These differences, expressed using a base 10 logarithm, are more clearly visualized in Figure 7.

Notably, MGDG species exhibited diverse patterns, with MGDG 32:1, MGDG 32:2, MGDG 34:1, MGDG 34:3 and MGDG 34:4 being significantly reduced in MM40 compared to MM0. Conversely, MGDG 34:2 was more abundant in MM40, indicating a preference for certain lipid species in response to distinct growth media. DGDG species also exhibited variability, with DGDG 32:1 and DGDG 34:3 displaying a remarkable increase in MM40, suggesting a potential role in adapting to this specific growth condition. Furthermore, TG species displayed a mixed response to the two media. While some TG species, such as TG 45:0, TG 52:2 and TG 55:0, were more abundant in MM40, others, including TG 42:0 and TG 47:0, showed higher levels in MM0. Similarly, SQDG and PG species exhibited distinct trends in their abundance, with some species being more prevalent in MM0 and others in MM40.

Lipids, such as MGDG and DGDG, showed significant increases or reductions in MM40. These modifications may represent cyanobacterial strategies to adapt to conditions, regulate membrane fluidity, or respond to environmental stimuli. TG, serving as energy reserves, also exhibited variations between MM0 and MM40. Additionally, the species of SQDG and PG displayed variations, indicating further regulation of lipid composition in response to specific environmental factors. These lipids can play both structural and functional roles, as highlighted in the literature [81]. For instance, a study on the cyanobacterium *Thermosynechococcus elongatus* revealed that several lipids are found in the photosystem II (PSII) and appear to have a role in the organization and functionality of this photosynthetic structure, with MGDG, DGDG, SQDG, and PG present around the D1/D2 reaction center of PSII [82,83]. Among these lipids, SQDG, an anionic lipid, has been shown to be particularly important for the growth and photosynthesis of *T. elongatus*. In fact, a mutant strain incapable of synthesizing SQDG experienced severe issues in the formation of PSI trimers and PSII dimers, as well as in energy transfer in the phycobilisomes [84].

Even though the biological significance of these lipids is not yet fully understood, glycolipids constitute a class of metabolites that have recently gained interest for their potential biotechnological applications. They are considered promising phytochemicals with a wide range of biological properties. [85,86,87,88]. For instance, glyceroglycolipids derived from algae act as immunosuppressants, reducing inflammation through the activation of regulatory T cells [89,90].

MGDG, DGDG, and SQDG have shown anti-inflammatory properties [91,92]. Other studies have highlighted the anti-inflammatory activity of MGDG on human joint cartilage and the inhibition of IL-8 production in the HT-29 cell line [93,94]. Additionally, there are indications of significant antiviral effects. For example, MGDG and DGDG inhibit the ability of *herpes simplex* virus 2 (HSV-2) to bind to cells and replicate in vivo [95,96]. Furthermore, MGDG has demonstrated antiviral activity and stimulates antibody production, contributing to influenza virus inhibition [97]. Other studies have shown that SQDG, isolated from algae, exhibits inhibitory effects on various viruses, including HSV-1, HSV-2, and HIV [97,98]. Additionally, recent research has highlighted the antibacterial activity of glyceroglycolipids. MGDGs have been found to have strong antibacterial activity against different species such as *Haemophilus influenzae*, *Legionella pneumophila*, *Streptococcus pyogenes*, *Clostridium difficile*, and *Staphylococcus aureus* [99]. On the other hand, SQDG has been discovered to inhibit the proliferation of *E. coli* cells [100]. The immunomodulatory, antiviral, and antibacterial properties of glyceroglycolipids could play a key role in maintaining the health and safety of astronauts during long-term space missions. During the latter ones, astronauts face a unique and challenging environment, exposed to microgravity, radiation, and other extreme conditions. In such circumstances, the astronauts’ immune system may be compromised, increasing the risk of infections and inflammations. The immunomodulatory properties of glyceroglycolipids could help the astronauts’ immune system, reducing the risk of inflammatory reactions and enhancing the body’s ability to defend against potential pathogens. Moreover, the antiviral and antibacterial activity of glyceroglycolipids could aid in preventing and treating infections during space missions, where the confined conditions may facilitate the spread of pathogens. Additionally, considering the limited resources available in space, the possibility of cultivating microalgae to obtain lipids and other essential nutrients could be an intriguing option to provide astronauts with a sustainable source of dietary supplements during long-duration missions.

### 3.5. Chlorophyll-a, Total Carotenoids, Phycocyanin and Allophycocyanin

**Figure 8:**
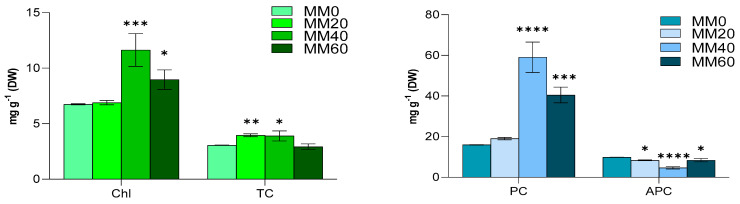
Effect of MM content on the pigment concentration (mg g^−1^ DW) after 43 days of cultivation. Chloropyll-a (Chl) and total carotenoids (TC) (**left**). Phycocyanins (PC) and allophycocyanins (APC) (**right**). Mean differences were tested using one-way ANOVA. No asterisk denotes *p*-value > 0.05; * *p*-value < 0.05; ** *p*-value < 0.01; *** *p*-value < 0.001; and **** *p*-value < 0.0001.

The feasibility of *C. thermalis* as a nutrient source for astronauts has been the subject of additional analysis. Pigment composition of the biomass was quantified, and the results are shown in Figure 5. It was observed that the utilization of MM40 resulted in an increase in total carotenoids (TC), chlorophyll-a (Chl), and, particularly, of phycocyanin (PC). The experiments demonstrated that the utilization of MM40 improved the content of antioxidant species in the produced biomass of *C. thermalis* with duplicate values for chlorophyll in MM40 ranging from 6.71 ± 0.05 mg g^−1^ to 11.65 ± 1.47 mg g^−1^ (Figure 8a). The TC content remains relatively constant at different concentrations of MM, ranging from 3.06 ± 0.02 to 3.90 ± 0.49 mg g^−1^, respectively, in MM0 and MM40. The content of PC almost quadrupled in the biomass of *C. thermalis* grown in MM40 (59.10 ± 7.46 mg g^−1^) when compared to MM0 (15.95 ± 0.12 mg g^−1^). The APC content showed an opposite trend (Figure 8b), halving to 4.71 ± 0.59 mg g^−1^ in the biomass from MM40 with respect to the one from MM0 (9.93 ± 0.07 mg g^−1^). Raw data used for Figure 8 are reported in Table S3 of Supplementary Materials.

In general, it can be stated that the use of MM40 induces an increase in the production of antioxidant species in *C. thermalis* biomass. This phenomenon can be attributed to both the content of metals present in MM40 as well as to higher content of urea, as a nitrogen source, in the synthetic urine used to prepare the MM. In the first case, the accumulation of metals from MM40 in cyanobacteria cells can lead to the formation of reactive oxygen species (ROS) such as hydroxyl radical (·OH), superoxide anion (O_2_^−^), singlet oxygen (O_2_*), and hydrogen peroxide (H_2_O_2_), which interact with lipids, proteins, and nucleic acids, causing oxidative stress. Among the defensive mechanisms are the synthesis of antioxidant compounds like pigments or enzymes (superoxide dismutase, catalase) responsible for neutralizing ROS and reducing metal ions into less reactive forms [101,102,103]. Regarding the effect of urea, it has been demonstrated that its use as a nitrogen source instead of inorganic nitrogen leads to an increase in both algal productivity and the content of chlorophyll and phycocyanin In *Spirulina platensis sp*. [104]. Consistently, Gladfelter et al. [105] discovered that, compared to nitrate, reduced nitrogen forms such as urea significantly promote algae growth and the accumulation of phycocyanin.

It should be noted that for the purpose of this work, a high level of antioxidants could help protect astronauts from oxidative stress arising from the exposition to ionizing radiation and microgravity taking place during both the interplanetary travel and the staying period on Mars. Moreover, cultivating cyanobacteria and microalgae with abundant antioxidants could preserve the cultures themselves from stress, enhancing their survival, productivity, and the quality of the biomass produced.

### 3.6. Antioxidant Activity

To determine the antioxidant power of the biomass produced by *C. thermalis* cultivated using MM40, the Trolox equivalent antioxidant capacity (TEAC) assay was used. The biomass grown in both media exhibited radical scavenging capacities that were comparable to those reported by Goiris et al. [106] for different microalgal strains. The biomass grown in both MM0 and MM40 exhibited radical scavenging capacities ranging from 17.83 ± 2.62 to 21.00 ± 0.47 mmol TEAC kg^−1^. These results demonstrate the potential of *C. thermalis* as a source of natural antioxidants. Interestingly, cultivation in MM40 resulted in an increase in antioxidant power by 17% if compared to that obtained with MM0. Such an increase could be ascribed to the induced biosynthesis of antioxidant compounds such as chlorophyll and phycocyanins, as a defense mechanism of the cells against the increased exposition to ROS. On the other hand, it is well known that microalgal cells synthesize antioxidant molecules under stress conditions [101,102,107,108,109]. Therefore, the increase in antioxidant power observed in MM40 could be a result of the induced stress response (due to high heavy metals, high salinity, and low nutrients in MM) in *C. thermalis* cells.

While the biochemical mechanisms underlying this result require further investigation, these findings suggest that the use of MM40 could enhance the antioxidant properties of the biomass produced with Martian regolith and urine.

### 3.7. Cytotoxicity of C. thermalis Extracts

**Figure 9:**
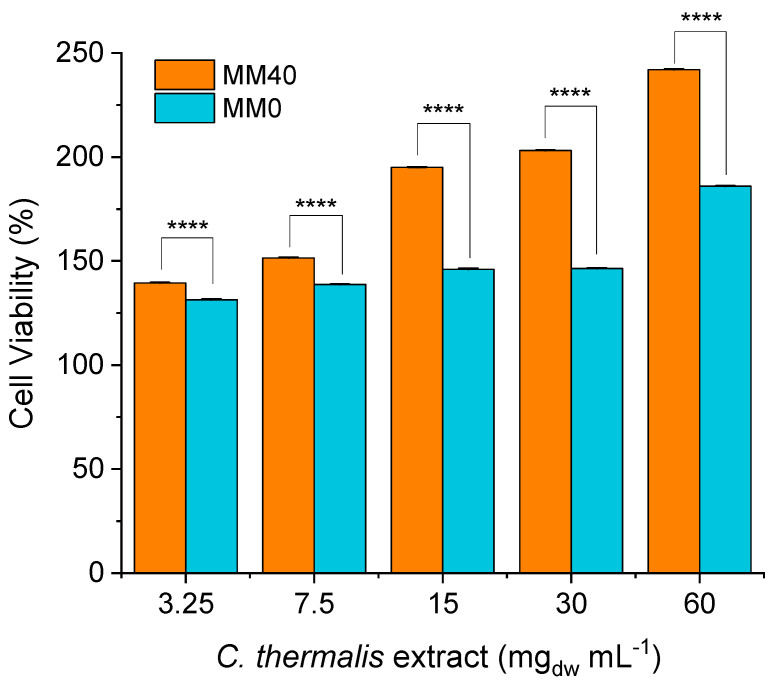
The effect of *C. thermalis* extract on HeLa cell viability showed no cytotoxic effects on HeLa cells. A comparison of groups was carried out using a 2-way ANOVA with Sidak’s multiple comparisons test. The significance level (alpha) was set at 0.05, and the sample size (n) for each group was 12. Mean differences were tested using two-way ANOVA. **** *p*-value < 0.0001.

To evaluate whether the biomass produced using Martian regolith leachate could exhibit toxic effects, a cytotoxicity test (MTT assay) of the extract obtained from *C. thermalis* was performed. Specifically, the extract was tested on HeLa cells, which are commonly used in cytotoxicity studies due to their high sensitivity to toxic compounds. The results showed that the extract from *C. thermalis* biomass previously cultured in both MM0 and MM40 did not significantly affect HeLa cell viability (Figure 9). The ability of *C. thermalis* to produce a non-cytotoxic biomass for human cells suggests that this cyanobacterium could be a safe and valuable source of nutrients and bioactive compounds for human consumption. The absence of observed cytotoxic effects supports the potential use of Martian regolith leachate and human urine as components of growth media for *C. thermalis*, as the resulting biomass would not contain harmful compounds. However, further studies are needed to confirm the safety and potential health benefits of consuming *C. thermalis* biomass.

Raw data used for Figure 9 are available in the Supplementary Materials (Table S4).

## 4. Conclusions

This study explored the potential of using *Chroococcidiopsis thermalis* as a sustainable nutrient and bioactive compound source for astronauts during long-duration space missions. The research aimed to optimize cultivation conditions using Martian regolith leachate and synthetic human urine. Results showed that replacing 40% of the conventional growth medium with a Martian regolith-based MM growth medium resulted in the best combination of biomass productivity and improved resource utilization on-site. The use of MM40 also promoted the production of antioxidant compounds, such as chlorophyll-a and phycocyanins, which protect cells from free radicals and oxidative stress, thus making *C. thermalis* more resilient under Mars-like stress conditions, as well as very interesting as a potential source of nutrients for astronauts’ consumption. The biomass of *C. thermalis* was found to be rich in essential macronutrients, including proteins, carbohydrates, and lipids, with an interesting lipid profile. Moreover, biomass cultivated with MM40 was confirmed to be safe for human consumption, as it was non-cytotoxic to human cells. These findings suggest that *C. thermalis* cultivated with MM40 could serve as a nutrient source and/or dietary supplement for astronauts, potentially reducing reliance on Earth supplies and enhancing the sustainability of space missions. However, further research is required to optimize cultivation conditions and thoroughly assess the impact on human health resulting from the consumption of *C. thermalis* biomass. Additionally, comprehensive studies are essential to investigate the presence of potential contaminants in *C. thermalis* biomass, including toxic molecules such as cyanotoxins. These investigations would contribute to establishing robust safety protocols and guidelines for the utilization of *C. thermalis* biomass in various applications, ensuring the mitigation of potential risks and the full realization of its benefits. Additionally, specific challenges related to the space environment, such as microgravity and radiation effects on this species, need further investigation. Comprehensive analysis will be crucial in developing optimal cultivation strategies and ensuring the safe and effective utilization of *C. thermalis* as a food resource for future space missions.

## Figures and Tables

**Table 1:** The concentration of macronutrients and micronutrients present in the Martian medium (MM).

Macronutrients		Micronutrients	
Component	(g × L^−1^)	Component	(mg × L^−1^)
Na_2_SO_4_	0.085	Al	2.4
C_5_H_4_N_4_O_3_	0.012	Ca	4.06
Na_3_C_6_H_5_O_7_ × 2H_2_O	0.036	Fe	3.205
C_4_H_7_N_3_O	0.044	K	4.16
CH_4_N_2_O	0.750	Mg	0.74
KCl	0.115	Mn	0.095
NaCl	0.087	Na	2.33
CaCl_2_	0.009	P	0.125
NH_4_Cl	0.063	Si	5.14
K_2_C_2_O_4_ × H_2_O	0.002	Ti	0.635
MgSO_4_ × 7H_2_O	0.054		
NaH_2_PO_4_ × 7H_2_O	0.146		
Na_2_HPO_4_ × 2H_2_O	0.041		

## Data Availability

Data is contained within the article or supplementary material.

## Supplementary Materials

The following supporting information can be downloaded at: https://www.mdpi.com/article/10.3390/life14020251/s1, Table S1: Final biomass productivity in exponential phase (g m^−3^ day^−1^); Table S2: Macronutrients content in *C. thermalis* cultivated with and without MM (%DW); Table S3: Pigment concentration (mg g^−1^ DW) after 43 days of cultivation; Table S4: Effect of *C. thermalis* extract on HeLa cell viability.

## Appendix A

**Table A1:** *C. thermalis* FAME composition.

FA	C:N	MM0 (DW%)	MM40 (DW%)	*p* Value
Caprylic acid	C8:0	0.64 ± 0.05	0.13 ± 0.02	****
Capric acid	C10:0	2.30 ± 0.04	2.50 ± 0.10	**
Lauric acid	C12:0	0.40 ± 0.02	0.40 ± 0.04	ns
Tridecanoic acid	C13:0	0.31 ± 0.01	0.28 ± 0.02	ns
Myristic acid	C14:0	0.80 ± 0.02	0.80 ± 0.04	ns
cis-10-Pentadecenoic acid	C15:1	0.90 ± 0.02	0.77 ± 0.03	ns
Palmitic acid	C16:0	10.40 ± 0.10	24.00 ± 0.1	****
Palmitoleic acid	C16:1	2.02 ± 0.02	3.07 ± 0.03	****
cis-Heptadecenoic acid	C17:1	0.11 ± 0.02	0.22 ± 0.02	ns
Stearic acid	C18:0	3.90 ± 0.05	3.90 ± 0.04	ns
(n-9) Oleic acid	C18:1	1.43 ± 0.03	3.70 ± 0.03	****
(n-6) Linoleic acid	C18:2	3.30 ± 0.05	1.80 ± 0.05	****
(n-6) γ-Linolenic acid	C18:3	1.06 ± 0.04	0.93 ± 0.03	ns
(n-3) α-Linolenic acid (ALA)	C18:3	0.80 ± 0.02	1.80 ± 0.01	****
Total saturates		18.7 ± 0.1	32.0 ± 0.2	****
Total monoenes		4.4 ± 0.1	7.7 ± 0.1	****
Total PUFA		5.10 ± 0.05	4.5 ± 0.1	****
S/U *		1.95	2.60	not tested

C:N refers to number of atom carbon (C) and double bonds (N). Data are expressed as a g/100g of dried biomass (mean ± SD). PUFA—polyunsaturated fatty acid; * Saturation ratio = (8:0 + 10:0 + 12:0 + 13:0 + 14:0 + 15:0 + 16:0 + 18:0)/(15:1 + 16:1 + 17:1 + 18:1 + 18:2 + 18:3 + 18:3). Mean differences were tested using two-way ANOVA. Not significant (ns) denotes that *p*-value > 0.05; * *p*-value < 0.05; ** *p*-value < 0.005; and **** *p*-value < 0.0001.

**Table A2:** List of exact mass measurement of the molecular ions and lipid species.

Lipid Species (C:N)	Fatty Acyl Chains in MS/MS Mode	Formula	Calculated *m*/*z*	Observed *m*/*z* of Molecular Ions	Error (ppm)
MGDG annotated as [M+NH_4_]^+^					
MGDG 31:2	15:1/16:1	C_40_H_72_O_10_	730.5464	730.5479	2.1
MGDG 32:1	16:0/16:1	C_41_H_76_O_10_	746.5777	746.5820	5.8
MGDG 32:2	16:1/16:1	C_41_H_74_O_10_	744.5620	744.5578	−5.6
MGDG 34:1	16:0/18:1	C_43_H_80_O_10_	774.6090	774.6065	−3.2
MGDG 34:2	16:0/18:2	C_43_H_78_O_10_	772.5933	772.5937	0.5
MGDG 34:3	16:0/18:3	C_43_H_76_O_10_	770.5777	770.5757	−2.6
MGDG 34:4	16:1/18:3	C_43_H_74_O_10_	768.5620	768.5610	−1.3
DGDG annotated as [M+NH_4_]^+^					
DGDG 32:1	16:0/16:1	C_47_H_86_O_15_	908.6305	908.6266	−4.3
DGDG 34:2	16:1/18:1	C_49_H_88_O_15_	934.6461	934.6433	−3.0
DGDG 34:3	16:0/18:3	C_49_H_86_O_15_	932.6305	932.6304	−0.1
DGDG 34:4	16:1/18:3	C_49_H_84_O_15_	930.6148	930.6147	−0.1
PC annotated as [M+H]^+^					
PC 32:1		C_40_H_78_NO_8_P	732.5538	732.5590	7.1
PC 32:2		C_40_H_76_NO_8_P	730.5381	730.5420	5.3
PC 36:2	18:1/18:1	C_44_H_84_NO_8_P	786.6007	786.6033	3.3
PC 36:4		C_44_H_80_NO_8_P	782.5694	782.5628	−8.4
TG annotated as [M+NH_4_]^+^					
TG 42:0	12:0/14:0/16:0	C_45_H_86_O_6_	740.6763	740.6810	−6.35
TG 44:0	14:0/14:0/16:0	C_47_H_90_O_6_	768.7084	768.7100	−3.12
TG 45:0	14:0/15:0/16:0	C_48_H_92_O_6_	782.7232	782.7235	−0.35
TG 46:0	14:0/16:0/16:0	C_49_H_94_O_6_	796.7389	796.7418	−3.64
TG 46:1	14:0/16:0/16:1	C_49_H_92_O_6_	794.7232	794.7260	−3.52
TG 47:0	15:0/16:0/16:0	C_50_H_96_O_6_	810.7545	810.7582	−4.56
TG 48:0	16:0/16:0/16:0	C_51_H_98_O_6_	824.7702	824.7750	−5.82
TG 49:0	16:0/16:0/17:0	C_52_H_100_O_6_	838.7858	838.7911	−6.32
TG 49:1	15:0/16:0/18:1	C_52_H_98_O_6_	836.7702	836.7750	−5.74
TG 50:1	16:0/16:0/18:1	C_53_H_100_O_6_	850.7858	850.7886	−3.29
TG 52:2	16:0/18:1/18:1	C_55_H_102_O_6_	876.8015	876.8047	−3.65
TG 54:0	18:0/18:0/18:0	C_57_H_110_O_6_	908.8641	908.8601	4.40
TG 54:3	18:1/18:1/18:1	C_57_H_104_O_6_	902.8171	902.8202	−3.43
TG 54:5	18:1/18:1/18:3	C_57_H_100_O_6_	898.7858	898.7809	5.45
TG 55:0	16:0/15:0/24:0	C_58_H_112_O_6_	922.8797	922.8784	1.41
SQDG annotated as [M-H]^-^					
SQDG 32:0	16:0/16:0	C_41_H_78_O_12_S	793.5141	793.5155	1.76
SQDG 32:1	16:0/16:1	C_41_H_76_O_12_S	791.4985	791.4999	1.77
SQDG 34:1	16:0/18:1	C_43_H_80_O_12_S	819.5298	819.5299	0.12
SQDG 34:2	16:0/18:2	C_43_H_78_O_12_S	817.5141	817.5140	−0.12
SQDG 34:3	16:0/18:3	C_43_H_76_O_12_S	815.4985	815.4993	0.98
PG annotated as [M-H]^−^ and as [M+NH_4_]^+^					
PG 34:2	16:1/18:1	C_40_H_75_O_10_P	745.5025/764.5436	745.5024/764.5474	−0.1/5.0
PG 34:3	16:0/18:3	C_40_H_73_O_10_P	743.4869/762.5280	743.4884/762.5313	2.1/4.3

Monogalactosyldiacylglicerol (MGDG), digalactosyldiacylglicerol (DGDG), phosphatidylcholine (PC), diacylglycerol (DG), triacylglycerol (TG), sulfoquinovosyldiacylglicerol (SQDG) and phosphatidylglycerol (PG) identified by RP-ESI-MS in positive and negative ion mode in the total lipid extracts of *C. thermalis* grown under different percentage of MM. When possible, the identification of the fatty acyl composition correspondent to each molecular ion was confirmed by the analysis of the LC-MS/MS spectra of each [M+CHO_2_]^−^ ion for PC. C is the total number of carbon atoms, while N the total number of double bonds. Mass measurement error (difference between the exact or theoretical mass and accurate mass) is expressed as parts per million (ppm).

**Table A3:** Normalized abundance of each lipid found in *Chroococcidiopsis thermalis* grown in different media and atmosphere.

Lipid Species (C:N)	MM-0	MM-40	*p* Value
MGDG 31:2	0.09 ± 0.01	0.09 ± 0.01	ns
MGDG 32:1	0.40 ± 0.06	0.07 ± 0.01	****
MGDG 32:2	0.34 ± 0.01	0.12 ± 0.02	***
MGDG 34:1	0.38 ± 0.03	0.09 ± 0.02	****
MGDG 34:2	0.07 ± 0.01	0.68 ± 0.03	****
MGDG 34:3	4.58 ± 0.36	0.07 ± 0.01	****
MGDG 34:4	2.68 ± 0.18	1.88 ± 0.02	****
DGDG 32:1	0.07 ± 0.01	0.30 ± 0.01	***
DGDG 34:2	0.06 ± 0.01	0.03 ± 0.01	ns
DGDG 34:3	0.03 ± 0.02	0.32 ± 0.03	****
DGDG 34:4	0.25 ± 0.03	0.08 ± 0.01	*
PC 32:2	0.09 ± 0.01	0.09 ± 0.01	ns
PC 36:2	0.02 ± 0.01	0.05 ± 0.01	ns
PC 36:4	0.02 ± 0.01	0.03 ± 0.01	ns
TG 42:0	0.37 ± 0.02	0.25 ± 0.03	ns
TG 44:0	0.28 ± 0.02	0.14 ± 0.04	ns
TG 45:0	0.14 ± 0.01	0.51 ± 0.07	****
TG 46:0	0.92 ± 0.04	1.05 ± 0.09	ns
TG 46:1	0.12 ± 0.02	0.06 ± 0.01	ns
TG 47:0	0.81 ± 0.08	0.31 ± 0.04	****
TG 48:0	0.08 ± 0.01	0.05 ± 0.01	ns
TG 49:0	0.11 ± 0.02	0.27 ± 0.04	*
TG 49:1	0.06 ± 0.01	0.03 ± 0.01	ns
TG 50:1	0.53 ± 0.04	0.57 ± 0.04	ns
TG 52:2	0.04 ± 0.01	0.21 ± 0.03	*
TG 54:0	1.22 ± 0.12	1.05 ± 0.08	*
TG 54:3	1.80 ± 0.1	1.94 ± 0.04	ns
TG 54:5	0.13 ± 0.01	0.15 ± 0.05	ns
TG 55:0	1.73 ± 0.01	2.13 ± 0.1	****
SQDG 32:0	0.10 ± 0.01	0.13 ± 0.04	ns
SQDG 32:1	0.14 ± 0.02	0.43 ± 0.09	****
SQDG 34:1	0.08 ± 0.01	0.03 ± 0.01	ns
SQDG 34:2	0.70 ± 0.09	0.48 ± 0.04	***
SQDG 34:3	0.83 ± 0.04	0.56 ± 0.08	****
PG 34:2	0.14 ± 0.03	0.14 ± 0.03	ns
PG 34:3	0.03 ± 0.01	0.04 ± 0.01	ns

Mean differences were tested using two-way ANOVA. Not significant (ns) denotes that *p*-value > 0.05; * *p*-value < 0.05; *** *p*-value < 0.001; and **** *p*-value < 0.0001.
